# Pearls and pitfalls in nonalcoholic fatty liver disease: tricky results are common

**DOI:** 10.20517/mtod.2021.02

**Published:** 2021-07-02

**Authors:** Brian J. Wentworth, Stephen H. Caldwell

**Affiliations:** Division of Gastroenterology & Hepatology, University of Virginia Health System, Charlottesville, VA 22908, USA.

**Keywords:** Nonalcoholic fatty liver disease, fibrosis, cryptogenic cirrhosis, sarcopenia, autoantibodies

## Abstract

Interpretation of diagnostic and surveillance laboratory results and imaging in liver disease is fraught with misinterpretation and/or uncertainty. Nonalcoholic fatty liver disease (NAFLD) represents an ever-growing proportion of liver disease cases but presents unique challenges for the clinician. Given the necessity of excluding other etiologies of liver disease, NAFLD can at times represent a challenging diagnosis as non-invasive assessment and biopsy are imperfect tests with important limitations. Similarly, cautious review of laboratory reports is necessary to avoid missing abnormal pathophysiology. The presence of lab values within the standard reference range may be concerning in the setting of chronic liver disease (“abnormally normal”) and conversely results flagged as abnormal may not necessarily be of great concern (“normally abnormal”). This review provides a framework for the clinician to review common diagnostic challenges in NAFLD and enhance patient care.

## INTRODUCTION

Liver disease is common in the United States and has been increasing across the world, driven by the obesity epidemic and corresponding rise in nonalcoholic fatty liver disease (NAFLD). Approximately 25% of the world population has NAFLD; up to 30% of these patients have steatohepatitis on biopsy (NASH) - leading to an estimated overall population prevalence of 5%−6%^[1]^. As liver disease progresses, ongoing inflammation stimulates hepatic stellate cells in the perisinusoidal space (space of Disse) to secrete excess collagen, tissue inhibitors of metalloproteinases, and matrix metalloproteinases. The sum of this activity leads to fibrosis and architectural remodeling, eventually culminating in loss of the normal lobular hepatic parenchyma in favor of discrete nodules surrounded by fibrous septa. Cirrhotic transformation occurs over many years in the majority of cases, yet its prevalence in the United States is 0.27% and is the twelfth leading cause of death overall^[2]^. Historically, the primary etiologies of cirrhosis have been alcohol and chronic hepatitis C, but NAFLD/NASH has now become the second leading indication for liver transplantation^[3]^. NAFLD is unique, however, as it is associated with the metabolic syndrome. Accordingly, the leading cause of death in patients with NAFLD (including cirrhosis) is cardiovascular disease (CVD), rather than liver-related complications, with degree of fibrosis on biopsy serving as an important independent predictor of mortality^[4]^.

To manage this increasing burden of liver disease from NAFLD, clinicians often utilize guidelines put forth by the American Association for the Study of Liver Diseases (AASLD), European Association for the Study of the Liver (EASL), Latin American Association for the Study of the Liver (ALEH), and the Asian Pacific Association for the Study of the Liver (APASL). Laboratory and imaging tests are frequently performed as part of the standard evaluation, yet much confusion exists. Liver disease, NAFLD not excepted, is fraught with interferences in homeostatic processes and there is significant heterogeneity between patients. This variance contributes to clinician discomfort (or mistaken confidence) with diagnostic algorithms and laboratory interpretation.

Although a comprehensive review of the diagnostic approach to liver disease and NAFLD in particular is beyond the scope of this review, we instead aim to provide clinicians a framework to better understand common diagnostic tests. We will also specifically review situations in which labs: (1) are within the normal reference range but fail to reflect a significant underlying physiology disturbance (“abnormally normal”); and (2) are outside the normal reference range but appropriately reflect expected physiologic disturbances in liver disease (“normally abnormal”).

## DIAGNOSTIC CONUNDRUMS IN NAFLD

### The role of non-invasive testing and liver biopsy in NAFLD

Liver biopsy remains the gold standard for the diagnosis of NAFLD and staging of fibrosis. However, it is an invasive procedure accompanying risks of bleeding, pneumothorax, or damage to nearby organs. In addition, biopsy is subject to sampling error as inflammation and fibrosis may be heterogenous in NAFLD patients^[5]^. A multitude of non-invasive tests are available in the clinician armamentarium, yet proper application and a thorough understanding of the limitations of these devices is paramount (see Table 1).

Assessment of steatosis aids in making the diagnosis of NAFLD, albeit it does not carry the same prognostic importance as defining the degree of fibrosis. Conventional ultrasound can assess for steatosis in a non-quantitative manner with significant interobserver variability, but evaluation for fibrosis is limited outside of surface contour nodularity. To improve the diagnostic ability of ultrasound, several techniques have been described to quantify the degree of steatosis, although they are not yet routinely employed in clinical practice. Most imaging devices today assume a constant speed of sound through the hepatic parenchyma at 1540 m/s (this is acceptable in healthy liver tissue only), which is problematic in fatty liver as there is a negative correlation between the amount of steatosis and speed of sound. Therefore, evaluating the speed of sound through the liver could provide an estimation of hepatic fat. Another technique described includes calculation of an attenuation coefficient (energy loss related to characteristics of a tissue medium, such as fat). Although there are several describe methodologies in the literature, perhaps the most commercially available and validated parameter is the controlled attenuation parameter (CAP) that is typically combined with VCTE (Fibroscan®, Echosens, Paris, France). Area under receiver operator curve values for determination of various degrees of steatosis when compared to histologic findings are good at 0.823–0.888, although not as reliable as liver biopsy or proton density fat fraction. Additionally, the accuracy of CAP in advanced fibrosis or cirrhosis is limited - thus, measurements should be interpreted cautiously in select patients^[6]^.

Defining the degree of fibrosis is important in patients with NAFLD as this is the principal determinant of the risk of progression to severe liver disease and more recent evidence suggests correlation with cardiovascular risk^[7,8]^. Several scoring systems using routinely acquired labs have been developed to estimate stage of fibrosis, which may help determine need for referral to hepatology clinic and/or liver biopsy. The most widely adopted tests include the NAFLD Fibrosis Score (NFS), Fibrosis-4 (FIB-4), and vibration-controlled transient elastography (VCTE; i.e., Fibroscan®). In the absence of an established diagnosis of diabetes, calculation of the homeostatic model assessment (HOMA; fasting insulin × fasting glucose/405) is required as part of the NFS for assessment of insulin resistance. Among biopsy-proved NAFLD patients, the FIB-4 carries an area under receiver operator characteristic curve of 0.86 with the NFS closely following at 0.81. Negative predictive values (NPV) for advanced fibrosis (F3-F4) are 95% and 92%, respectively, which some authors argue may allow a majority of NAFLD patients to forgo biopsy^[9]^. However, recent literature suggests a more nuanced use of these scores is needed.

The NFS and FIB-4 were originally validated in patients aged 35–65 (with the majority of patients in the 45–55-year age range) but tend to perform poorly in older patients (age > 65 years). Cutoffs of 0.675 and 2.67, respectively, for the presence of advanced fibrosis should be adjusted downward to 0.12 and 2.0 in this older cohort to improve specificity without significantly degrading sensitivity^[10]^. The utility of NFS and FIB-_4_ also diminishes in younger patients (age < 35), although specific cut-offs are less well-established given that the presence of advanced fibrosis is less common^[10,11]^. Additionally, as age is a significant component to these non-invasive tests, many patients will have indeterminate range scores necessitating further workup. Nonetheless, these tests are easily performed in the office and have high utility as a screening mechanism in the primary care setting to triage necessity of hepatology referral and/or additional workup^[12]^.

A full review of the clinical scenarios in which VCTE may be employed is beyond the scope of this manuscript, but it can be a useful tool to confirm hepatic steatosis and more importantly, estimate degree of fibrosis in NAFLD. Clinicians should also be aware that the liver stiffness measurements (LSM) provided by VCTE, measured in kilopascals (kPA), vary depending on the disease etiology. Tapper *et al*.^[13]^ determined that a kPA < 7.9 had 100% NPV for advanced fibrosis in a population of biopsy-proved NAFLD patients, with an optimal cut off of 9.9 kPA providing 95% sensitivity and 77% specificity. However, adequate performance of VCTE is limited by several factors. Hepatic congestion, such as from heart failure or the post-prandial state (hence the minimum 3 h fast required before VCTE), extrahepatic cholestasis, severe hyperbilirubinemia, operator inexperience, and obesity may preclude accurate readings^[14]^. Implanted electrical devices, such as pacemakers or defibrillators, negate the ability to perform VCTE. Nonetheless, Eddowes *et al*.^[15]^ recently published reassuring data suggesting that the degree of steatosis and probe type did not affect LSM in NAFLD. This confirms prior literature validating the use of the XL probe in obese patients and/or those patients in whom reliable LSM measurements are unable to be obtained with the standard M probe^[16]^.

Other available forms of elastography include proton shear wave elastography (pSWE), 2-dimensional shear wave elastography (2D-SWE), and magnetic resonance elastography (MRE). Accuracy of pSWE and 2D-SWE are similar to VCTE but limitations include obesity, a significant percentage of unreliable scans, and high interobserver variability (although improves with experience). Although MRE is costly and requires radiologic expertise which have limited its widespread adoption, its strengths include examination of whole liver sections (thus mitigating sampling error), low technical failure rates (< 5%), and high area under receiver operator curve (> 0.90) for detection of stage 2 and above fibrosis. While massive ascites can limit signal detection, MRE is less limited by obesity as compared to VCTE, pSWE, and 2D-SWE^[17]^.

At least two non-invasive tests are typically employed in the workup of NAFLD. Given the commonality of non-invasive biomarker scores and VCTE, a newer score called the FAST score incorporating the AST level and VCTE parameters has been designed and validated in an international cohort to predict patients at risk for NASH and progression of fibrosis^[18]^. However, liver biopsy may ultimately be necessary in select patients. The current AASLD practice guidance suggests its use if alternative etiologies of hepatic steatosis or chronic liver disease cannot be excluded, as well as if steatohepatitis or advanced fibrosis may be present - particularly if it may influence therapeutic decisions and affect patient prognosis^[19]^. Similarly, it is also common in clinical practice to utilize biopsy when there are discordant non-invasive tests, indeterminate tests, and to restage NAFLD/NASH every several years.

### NAFLD in non-obese patients

While the plurality of patients with NAFLD are obese, a substantial proportion are either non-obese (but overweight) or lean (non-overweight). A recent meta-analysis revealed that the prevalence of non-obese and lean NAFLD was 41% and 19%, respectively, within the overall NAFLD population. Perhaps more striking is that 39% of these patients had NASH and 29% had significant fibrosis^[20]^. Despite these alarming numbers, NAFLD is often asymptomatic and there is substantial delay in diagnosis in non-obese patients. Interestingly, non-obese NAFLD patients typically have signs of an unhealthy metabolic profile, including dyslipidemia, insulin resistance, and hypertension - all of which are independently associated with the development of NAFLD. Additionally, there is accumulating evidence that the presence of these derangements in non-obese individuals is associated with worse liver histology and cardiovascular outcomes than obese patients without an unhealthy metabolic status^[21]^.

Early identification of these non-obese or lean patients is necessary and the diagnosis is often made after performance of an otherwise unrevealing workup for elevated liver enzymes or the incidental discovery of steatosis on imaging. Calculation of HOMA (see prior section) can be helpful to identify insulin resistance, as well as checking a fasting lipid panel. Non-invasive evaluation with various scoring systems, liver fibrosis markers, or VCTE can also be undertaken, as described in the prior section. However, if the etiology of steatosis and/or degree of fibrosis is unclear based on history and initial workup alone, liver biopsy may provide additional insight. Issues with adequacy of sample, patchiness of disease, and interobserver variation in steatosis and fibrosis scores are important limitations of biopsy which can affect interpretation^[22]^.

### Hepatocellular carcinoma screening

The incidence of hepatocellular carcinoma (HCC) has been increasing for the past several decades and is now the fifth most common cause of cancer worldwide and third leading cause of cancer-related death^[23]^. While HCC most commonly arises in the setting of cirrhosis from any etiology, it also may be seen in the absence of cirrhosis in some conditions. A recent meta-analysis showed that non-cirrhotic NASH patients have an increased risk of HCC compared to non-cirrhotic patients with other etiologies of chronic liver disease^[24]^. However, the current AASLD guidance statement only recommends screening for HCC in patients with cirrhosis (of any etiology) and certain populations of patients with non-cirrhotic chronic hepatitis B. While this statement recognizes the increased risk of HCC in non-cirrhotic patients with NASH, the benefit and cost-effectiveness of screening remains uncertain given lack of data and the large number of patients potentially at risk^[25]^.

Semiannual ultrasound (US) with or without measurement of alpha fetoprotein (AFP) is recommended as the first-line screening test for HCC across all etiologies of liver disease and cirrhosis. While US is widely available and inexpensive, up to 20% of exams are considered inadequate, particularly in patients who are obese, use alcohol, or have NAFLD^[26]^. Several strategies may be implemented to counter the diagnostic limitation of US. First, contrasted cross-sectional imaging can overcome many of the technical factors preventing adequate performance of US and has the added benefit of better characterizing hepatic lesions. Multiphase CT or MRI are the preferred modalities, with selection best determined by the clinician based on patient and local expertise factors^[25]^. Second, the non-invasive GALAD scoring system has more recently been introduced as an alternative method to try to identify HCC earlier in patients with NASH, including those without cirrhosis. The score is an acronym derived from its components - gender, age, AFP-L3, AFP, and des-gamma carboxyprothrombin. In a recent hybrid study involving a retrospective case-control design which was then prospectively validated in a different cohort, the GALAD score provided high sensitivity and specificity and was able to identify patients who developed HCC over a year before diagnosis, including non-cirrhotics^[27]^. While these results are promising, further validation in larger, multicenter fashion is needed.

### Cryptogenic cirrhosis - burnt-out NASH by another name?

Historically, cryptogenic cirrhosis (CC) has accounted for between 5%−30% of all cases of cirrhosis^[28]^. However, improved diagnostic capabilities over time and confirmation of the association between metabolic risk factors and the development of NAFLD/NASH have allowed clinicians to better ascertain the etiology of cirrhosis. An early study of 70 patients with CC demonstrated that many of the cases shared risk factors of known NASH patients, including type 2 diabetes mellitus and obesity. Interestingly, these cryptogenic patients often were older females. The combination of older age, metabolic risk factors, and bland biopsies (no evidence of chronic viral hepatitis, autoimmune, or metabolic liver disease features) led the authors to hypothesize that in fact a substantial portion of diagnoses may be able to be reclassified as NASH since loss of hepatic fat and steatohepatitis occur over time in NASH patients^[28]^.

While a recent retrospective study investigating the United Network for Organ Sharing database proposed that CC may indeed be a distinct entity from NASH cirrhosis^[29]^, several methodologic issues temper these findings^[30]^. Further evidence that CC and NASH cirrhosis may be similar entities was shown in a study of 83 liver transplant recipients (46 with NASH and 37 with CC). Twenty patients had histologically confirmed recurrence of NASH (15 with NASH and 5 with CC as the original etiology of liver disease) at a mean time of 18 months post-transplant. This group had very high rates of metabolic syndrome and insulin use before transplant, prompting the authors to advocate for targeted metabolic interventions to prevent NASH recurrence, since 30% of patients ultimately required re-transplantation^[31]^.

Given the ubiquity of serologic testing, non-invasive modalities, and performance of liver biopsy in the evaluation of liver disease and staging of hepatic fibrosis, the term CC should be used sparingly. A thorough history should be obtained from the patient, including alcohol intake (presently and over time), comorbid conditions, any prior history of excess weight/obesity (as patients with decompensated cirrhosis may have lost weight), and family history of liver disease. Although in the strict sense NAFLD may only be diagnosed when there is ≥ 5% steatosis (either by imaging or biopsy) and exclusion of significant alcohol use^[19]^, clinicians may reasonably assume it is the underlying etiology a patient’s liver disease in the right clinical context.

## ABNORMALLY NORMAL LABS: AN EASY OVERSIGHT

### Aminotransferases

Asymptomatic elevations in liver enzymes, particularly the aminotransaminases, often are a presenting feature of NAFLD/NASH. Nonetheless, normal aminotransferase levels should not reassure patients or clinicians, as they do not independently predict histologic findings or identify risk of progression to fibrosis^[32]^. Alanine aminotransferase (ALT) levels can be within normal limits anywhere along the NAFLD spectrum, ranging from simple steatosis to cirrhosis. Furthermore, patients with normal ALT and NAFLD share similar demographic and risk factors when compared to those with abnormal ALT levels^[33]^. Thus, serial measurement of liver enzymes in isolation is unhelpful when following NAFLD patients longitudinally since they do not predict progression of fibrosis. However, their inclusion in validated non-invasive scoring systems is acknowledged and when combined with other clinical parameters (i.e., serum tests, imaging, VCTE, and/or liver biopsy), may help inform management and predict prognosis.

Another important point for clinicians to note is that reference ranges for normal transaminase levels vary by laboratory. Therefore, values reflective of subtle underlying hepatic inflammation may not be automatically flagged as abnormal. Although the thresholds representing truly normal levels are subject to some debate, the most widely accepted upper limit for ALT in men is 30 IU/L and 19 IU/L for women, derived from an Italian blood donor cohort^[34]^. However, other authors argue that this is overly sensitive and would classify approximately 40% of the US population as abnormal. If not being used to identify hepatitis C-infected individuals, thresholds of 44 IU/L for men and 32 IU/L for women may be suitable alternatives. Use of these cut-offs would reduce the proportion of the US population with abnormal transaminase levels to 11%^[35]^.

Additionally, aminotransferase levels are known to vary between patients based on demographic or underlying conditions. Obese patients tend to have higher levels; ALT levels may decline with weight loss. Aspartate aminotransferase (AST) is known to be 15% higher at baseline in African-American men and an inherent defect in enzymatic clearance which is otherwise clinically insignificant may lead to asymptomatic elevations^[36]^. Therefore, clinicians need to interpret liver enzymes with caution (see Table 2) as there is no specific ALT level which predicts NASH and/or advanced fibrosis^[37]^.

### Creatinine

Renal dysfunction commonly accompanies liver disease, particularly in patients with cirrhosis. Current clinical estimates of renal function rely on the measurement of serum creatinine. The liver synthesizes its precursor, creatine, which is then stored in skeletal muscle and finally excreted by the kidneys as creatinine. In non-diseased individuals, there is a fairly stable total body content of creatine thus creatinine excretion approximates the glomerular filtration rate (GFR), although it does not account for the small amount of creatinine secreted by renal tubular cells.

Reliance on serum creatinine and traditional formulas such as the Modification of Diet in Renal Disease (MDRD), Cockcroft-Gault, and Chronic Kidney Disease Epidemiology Collaboration is highly problematic in patients with cirrhosis (see Table 2). Several inherent conditions contribute to low serum creatinine levels, including decreased hepatic synthesis of creatine, ascended tubular secretion of creatinine, and sarcopenia^[38]^. Accordingly, a 24 h creatinine clearance is unlikely to provide any additional benefit over spot estimations based on a basic chemistry panel with the aforementioned formulas. Inulin clearance is considered the gold standard for measurement of GFR but is not routinely available in clinical practice. More recent literature proposes that measurement of serum cystatin C, a biomarker of kidney injury influenced less by extrarenal factors such as gender, age, inflammation, or muscle mass, may have utility in assessing renal function in patients with cirrhosis^[39]^. A Korean study by Yoo *et al*.^[40]^ noted that the MDRD-eGFR overestimated eGFR in female patients, sarcopenic males, and patients with decompensated cirrhosis (Child-Pugh classification B or C). The same study also concluded that cystatin C-based estimation of GFR correlated better with true GFR and was superior to MDRD-eGFR in predicting overall survival and incidence of acute kidney injury^[40]^. Nonetheless, one must be cautious when interpreting these results, given the MDRD was derived from an American population of Caucasian and African-American patients, who at baseline have higher muscle mass than Asian patients^[39]^.

While sarcopenia is well-known to occur in the setting of cirrhosis, it also has been associated with NAFLD, including NASH and advanced fibrosis^[41–43]^. Interestingly, this association appears independent of obesity and/or insulin resistance^[42,43]^. As NAFLD has become the second leading indication for liver transplantation in the United States, the importance of recognizing sarcopenia and inaccurate estimates of renal function becomes paramount for clinicians. The current liver transplantation allocation system relies on the Model for End-Stage Liver Disease score, of which creatinine is an important component. Similarly, the guidelines to quality for simultaneous kidney transplantation also rely in part on accurate estimation of GFR^[44]^. Thus, certain patients may be disadvantaged (including women and sarcopenic men) through chronic underestimation of their need for immediate organ access.

### Hemoglobin A1c

Measurement of impaired fasting glucose and/or diabetes can be achieved through several different modalities. However, perhaps the mostly widely and easiest to use clinically is assessment of glycated hemoglobin (hemoglobin A1c) as it is less susceptible to temporary fluctuations in glycemia secondary to diet, exercise, or illness. Up to 80% of patients with chronic liver disease are known to have insulin resistance, including most patients with NAFLD^[45,46]^. While hemoglobin A1c is routinely measured in these patients, its accuracy falls in patients with decompensated cirrhosis, particularly those that are severely anemic^[45]^.

Hemoglobin A1c levels also need to be interpreted with caution in other scenarios. In iron deficiency anemia, the most common cause of anemia worldwide, a paradoxical increase in A1c levels may be seen secondary to elongation of erythrocyte lifespan. However, in other forms of anemia, particularly hemolytic, A1c levels may be low secondary to decreased erythrocyte lifespan^[47]^. Considerred the limitations of A1c measurement, clinicians should discreetly interpret values in patients with liver disease and/or anemia to avoid potentially harmful adjustments (or lack thereof) of antihyperglycemic therapy (see Table 2).

## AFP

The AASLD Practice Guidance on HCC allows clinicians to consider measurement of alpha-fetoprotein along with US screening^[25]^. A threshold of 20 ng/mL is typically considered positive and requires further evaluation, although the positive predictive value remains low at only 25%. Nonetheless, it is frequently ordered with the intent of increasing ultrasound sensitivity, as a recent meta-analysis supported its utility in detecting early HCC^[48]^. Other literature has demonstrated its practicality in longitudinal fashion^[49]^.

However, AFP may not be elevated in up to 30% of patients with HCC, a subgroup colloquially known as “non-secreters”. Clinicians should not use AFP alone for HCC screening nor should further evaluation of an indeterminate or high-risk liver lesion on US be deferred if the AFP is normal (see Table 2). Patients should also be aware that the prognosis of a non-AFP secreting HCC is not substantially different from an AFP-secreting tumor and post-treatment surveillance similarly consists of serial abdominal and often chest imaging.

## CAUTIOUS INTERPRETATION REQUIRED: NORMALLY ABNORMAL LABS IN NAFLD

### Platelets

There is accumulating evidence that thrombocytopenia itself can be seen before the development of advanced fibrosis and thus may be an independent manifestation of the NAFLD disease process (see Table 3). In two recent small cohorts, the prevalence of thrombocytopenia in non-cirrhotic NAFLD was found to be 24%−28% and was associated with increased body weight^[50,51]^. These studies though defined NAFLD based on non-invasive measures; a larger retrospective Brazilian study of 441 patient with biopsy-proven NAFLD reported a lower prevalence of thrombocytopenia in non-cirrhotic patients at 3.2%^[52]^. Nonetheless, whether this mild thrombocytopenia is clinically important remains unclear as rates of bleeding events or need for treatment do not differ from patients with normal platelet levels^[50]^.

Despite its potential presence in NAFLD before the development of significant portal hypertension, splenomegaly, and/or cirrhosis, thrombocytopenia remains most common in these latter scenarios. Its presence should immediately prompt the clinician to perform fibrosis staging given this is known significant predictor of advanced fibrosis in NAFLD^[53]^. Consequently, platelet count is an important component of several non-invasive measures of fibrosis described previously (i.e., NFS, FIB4, *etc*.).

### High-density lipoprotein

The liver is the principal organ involved in cholesterol homeostasis and regulates the release of very-low density lipoproteins and high-density lipoprotein (HDL), as well as the uptake of low-density lipoproteins, intermediate-density lipoproteins, chylomicron remnants, and HDL^[54]^. While the pathophysiology of normal cholesterol metabolism is complex and beyond the scope of this review, it is important to understand that several of cholesterol pathways are dependent on hepatically synthesized enzymes. One of the key enzymes is lecithin-cholesterol acyltransferase (LCAT), whose activity allows esterification of free cholesterol and formation of larger (more mature) HDL particles.

In NAFLD, patients accumulate free cholesterol within the liver, stimulating Kupffer and stellate cells to induce steatohepatitis and fibrosis (lipotoxicity)^[55]^. The resulting steatosis impairs insulin signaling, contributing to insulin resistance and increasing the risk of developing type 2 diabetes mellitus^[56]^. A typical lipid panel in NAFLD often demonstrates high triglycerides, low HDL, and high LDL - a pattern well known to promote cardiovascular atherosclerosis. In particular, low levels of HDL increase the risk of CVD through multiple mechanisms including but not limited to promoting efflux of cellular cholesterol, reducing inflammation, decreasing platelet aggregation, and improving glucose metabolism^[57]^. While low HDL levels are common in NAFLD, CVD risk is both heightened and more severe independently of other established risk factors^[56]^.

Low HDL levels are also common in patients with decompensated cirrhosis of both NAFLD and non-NAFLD etiology (see Table 3). This phenomenon is at least partly driven by a decrease in LCAT activity^[58,59]^ and is a poor prognostic sign. A 2003 study by Habib *et al*.^[54]^ found that low HDL levels were predictive of 6- and 12-month mortality independent of the MELD score; levels < 30 mg/dL in particular were associated with a three-fold increase in pre-transplant mortality. Low HDL levels in patients with decompensated cirrhosis also are associated with relative adrenal insufficiency^[60]^, a multifactorial endocrinologic dysfunction that may represent an impaired stress response rather than absolute glucocorticoid deficiency and independently predictive of mortality^[61,62]^.

### Autoantibodies

The diagnosis of NAFLD (or NASH) can only truly be made after exclusion of all other etiologies of liver disease based on serologic and histologic assessment. Autoantibodies are commonly attained as part of the standard smorgasbord of labs ordered to evaluate conditions such as autoimmune hepatitis, primary biliary cholangitis, primary sclerosing cholangitis, or an overlap syndrome. However, these conditions are far rarer than NAFLD and thus without an appropriate clinical context, autoantibodies may be positive in non-autoimmune conditions. Autoantibodies are seen in 20%−35% of patients with NAFLD^[63,64]^, with antinuclear antibodies (ANA) being most common (13%−21%)^[63–66]^. Smooth muscle antibodies (SMA) may also be found in 3%−5% of NAFLD patients; the combination of ANA and SMA positivity is well-documented but less common. The prognostic value of autoantibodies in NAFLD remains unclear, as there is conflicting evidence in the literature regarding an association with worsened histologic features (inflammation, fibrosis) or insulin resistance. Nonetheless, it is important to avoid diagnosing a patient prematurely with an autoimmune liver disease based solely on autoantibody positivity - patient and family history, degree of autoantibody titer, and additional serologic workup all play an important role in helping define the etiology of liver disease. Liver biopsy may be necessary if there is clinical concern for an autoimmune liver disease as the treatment for these conditions differs vastly from that of NAFLD/NASH, including potential medication-related toxicity or unnecessary immunosuppression (see Table 3).

### Ferritin

Ferritin is the primary iron storage protein of the liver and may be elevated in the setting of primary or secondary states of iron overload. However, ferritin is an acute phase reactant and thus is frequently elevated in inflammatory states^[67]^ including excessive alcohol use, the metabolic syndrome^[68]^, and NAFLD^[69]^. Regarding the latter, hyperferritinemia has been associated with worse histologic activity and is an independent indicator of both steatosis and advanced fibrosis^[69,70]^. The cytological changes are thought to be a result of ferritin acting as a proinflammatory cytokine with downstream activation of hepatic stellate cells and stimulation of fibrogenesis pathways in an iron-independent fashion^[71]^. Interestingly, elevated ferritin levels also carry prognostic value among patients listed for transplant, including NAFLD and non-NAFLD etiologies of liver disease. In this population, a ferritin level of at least 200 μg/L has been associated with the development of portal hypertension-related decompensations, spur cell anemia, and hepatorenal syndrome, in addition to increased 6-month and 1-year mortality^[72]^.

Like autoantibodies, serum ferritin levels are commonly obtained in the workup of elevated liver enzymes or cirrhosis. Given their ubiquity and lack of specificity, high ferritin levels should be interpreted with caution. In the setting of normal transferrin saturation, hereditary hemochromatosis (HH) is unlikely but further evaluation for HH with HFE gene analysis is indicated if the transferrin saturation is ≥ 45%^[73]^. Clinicians must be aware that while HH is common, there is significant phenotypic variation given incomplete penetrance. Clinically relevant iron overload occurs in patients who are homozygous for the *C282Y* gene; C282Y/H63D compound heterozygotes typically require a second insult (such as alcohol use or NAFLD) to manifest hepatic fibrosis^[73]^. As metabolic risk factors often coexist in patients with elevated ferritin levels, NAFLD should be considered as either a competing or concurrent diagnosis to HH, with liver biopsy necessary to distinguish relative contributions (see Table 3).

### Vitamin B_12_

Anemia is common in liver disease, particularly in cirrhosis. A multifactorial etiology is typically present, including but not limited to hypersplenism, chronic bone marrow suppression, acute and chronic gastrointestinal bleeding, and/or hemolysis if spur cells are present^[74,75]^. Macrocytosis is well known to occur in the setting of alcohol abuse, however it also can result from chronic malnutrition and/or malabsorption in patients with chronic liver disease. Folic acid and vitamin B_12_ (cobalamin) deficiency are common entities but diagnosis of the latter can be more challenging in the setting of liver disease. Elevated serum vitamin B_12_ levels are seen in 7%−18% of hospitalized patients and do not necessarily exclude underlying deficiency^[76]^. An imbalance in B_12_ plasma binding proteins (haptocorrin, transcobalamin) secondary to increased synthesis or decreased clearance typically explains these high B_12_ levels. Specific to liver disease, elevated B_12_ levels result from abnormal hepatic clearance of haptocorrin and release of stored B_12_ from damaged hepatocytes (see Table 3); concurrent renal dysfunction may also contribute through poor B_12_ clearance^[76,77]^. Therefore, in the setting of liver disease, methylmalonic acid (MMA) should be measured instead, with elevated levels suggestive of B_12_ deficiency (cobalamin serves a cofactor for the conversion of MMA to succinyl coenzyme A)^[78]^.

Vitamin B_12_ deficiency is likely underdiagnosed. Its prevalence in the general population rises with age, estimated at 6% in patients less than 60 years and up to 20% in patients above the age of 60^[79]^. Surprisingly, autoimmune gastritis causing pernicious anemia is not frequently seen with autoimmune hepatitis^[80]^. However, patients with concurrent NAFLD and diabetes may be at increased risk for B_12_ deficiency^[81]^ as long-term use of metformin can induce the condition. Although clinical guidelines around this topic are lacking, it may be prudent for clinicians to consider screening patients who have been on therapy for at least a year with measurement of MMA, as it takes between 1–5 years to deplete hepatic B_12_ stores^[81]^.

## CONCLUSION

In summary, this review describes challenging areas in the diagnosis and management of NAFLD. In particular, close scrutiny of laboratory results is required as labs which are “abnormally normal” and “normally abnormal” may coexist in the same patient. Clinicians who realize these intricacies will be enabled to provide excellent patient care and prevent unnecessary additional testing, procedures, or potentially harmful therapies.

## Figures and Tables

**Table 1.** Assessment of NAFLD diagnostic modalities

Diagnostic test	Pros	Cons
NAFLD fibrosis score (NFS)	• Non-invasive • Easy to obtain parameters and calculate • Useful at extremes to rule-out or rule-in advanced fibrosis • May allow primary care providers to triage need for hepatology consultation	• Less reliable with ages < 35 or > 65 • Large range of indeterminate values necessitating further evaluation
FIB-4 score	• Non-invasive • Easy to obtain parameters and calculate • Useful at extremes to rule-out or rule-in advanced fibrosis • May allow primary care providers to triage need for hepatology consultation	• Less reliable with ages < 35 or > 65 • Large range of indeterminate values necessitating further evaluation
Ultrasound		
Conventional	• Non-invasive • Widely available, inexpensive • Evaluates liver morphology, splanchnic vessel patency, spleen size	• Operator dependent • Estimation of steatosis is subjective • Adequacy limited by habitus
Quantitative	• Quantifies degree of steatosis • Controlled attenuation parameter (CAP) reported along with liver stiffness measurement (LSM) on VCTE*	• Less widely available • Does not quantify fibrosis
Elastography		
VCTE*	• Non-invasive • Inexpensive • Useful at extremes to rule-out or rule-in advanced fibrosis • Quantify steatosis (CAP)	• Requires specialized training to perform and interpret • Examination adequacy limited by habitus • Biopsy may still be required if discordant results with clinical picture or to confirm advanced fibrosis
pSWE/2D-SWE	• Similar accuracy to VCTE	• Obesity may increase likelihood of unreliable examination • High interobserver variability
MRE	• Whole liver estimation (minimal sampling error) • Sensitive for stage 2–4 fibrosis • Improved accuracy in obese individuals as compared to other modalities	• Expensive • Requires special expertise to interpret • Massive ascites limits accuracy
Biopsy	• Widely available • Gold standard for histologic assessment and necessary to diagnose NASH (*vs*. NAFLD)	• Invasive, risk of complications • Sampling error • Requires pathology expertise

* Most prominent device on market = Fibroscan® (Echosens, Paris, France). VCTE: Vibration controlled transient elastography; pSWE: proton shear wave elastography; 2D-SWE: 2-dimensional shear wave elastography; MRE: magnetic resonance elastography; FIB-4: fibrosis-4; NAFLD: nonalcoholic fatty liver disease.

**Table 2.** ‟Abnormally normal” labs in NAFLD

Laboratory test	Comments
Aminotransferases (AST, ALT)	• AST not specific for liver dysfunction^*^ • Not well-correlated with histologic findings • Normal values vary by reference lab^†^, race, weight
Creatinine	• Estimated GFR (eGFR) problematic in non-Caucasian or African-American patients • eGFR may overestimate renal function in women and patients with sarcopenia
Hemoglobin A1c	• Accuracy impaired in patients with decompensated cirrhosis • Can be falsely elevated (iron-deficiency) or decreased (hemolytic) in anemic states^‡^
Alpha fetoprotein (AFP)	• Low PPV at common threshold of 20 ng/mL • Up to 30% of HCC tumors are non-secreters

* AST also found in skeletal and cardiac muscle, red blood cells, bone.

† Widely accepted upper limit of normal = 30 IU/L for men and 19 IU/L for women.

‡ Related to erythrocyte lifespan. AST: Aspartate aminotransferase; ALT: alanine aminotransferase; GFR: glomerular filtration rate; PPV: positive predictive value; HCC: hepatocellular carcinoma; NAFLD: nonalcoholic fatty liver disease.

**Table 3.** ‟Normally abnormal” labs in NAFLD

Laboratory test	Comments
Platelets	• Most commonly develops with advanced fibrosis and portal hypertension in all etiologies of liver disease • Can be seen in NAFLD before development of above
High-density	• Low levels can be seen as part of metabolic syndrome in NAFLD and are common in decompensated cirrhosis
lipoprotein	• Low levels portend poor prognosis and are associated with adrenal dysfunction in decompensated cirrhosis
Autoantibodies	• Seen in 20%−35% of NAFLD patients • ANA > > ASMA^*^; unclear if changes prognosis • If concern for possible autoimmune liver disease, requires biopsy to differentiate from NAFLD
Ferritin	• Hyperferritinemia associated with worse histologic activity, portal hypertension-related decompensations, hepatorenal syndrome, and mortality • Markedly elevated levels, particularly if % saturation is high, require HFE genetic testing to rule out hereditary hemochromatosis
Vitamin B_12_	• Levels elevated in both acute and chronic liver disease secondary to abnormal clearance of the plasma binding protein haptocorrin and release of stored B_12_ from hepatocytes • Measurement of methylmalonic acid (MMA) is necessary to assess for deficiency in liver disease

* ANA positive in 13%−21% and ASMA positive in 3%−5% of NAFLD patients. ANA: Antinuclear antibody; ASMA: anti-smooth muscle antibody; HFE: homeostatic iron regulator gene; NAFLD: nonalcoholic fatty liver disease.
